# Jacobsen Catalyst as a Cytochrome P450 Biomimetic Model for the Metabolism of Monensin A

**DOI:** 10.1155/2014/152102

**Published:** 2014-05-28

**Authors:** Bruno Alves Rocha, Anderson Rodrigo Moraes de Oliveira, Murilo Pazin, Daniel Junqueira Dorta, Andresa Piacezzi Nascimento Rodrigues, Andresa Aparecida Berretta, Ana Paula Ferranti Peti, Luiz Alberto Beraldo de Moraes, Norberto Peporine Lopes, Stanislav Pospíšil, Paul Jonathan Gates, Marilda das Dores Assis

**Affiliations:** ^1^Departamento de Química, Faculdade de Filosofia, Ciências e Letras de Ribeirão Preto, Universidade de São Paulo, 14040-901 Ribeirão Preto, SP, Brazil; ^2^Departamento de Análises Clínicas, Toxicológicas e Bromatológicas, Faculdade de Ciências Farmacêuticas de Ribeirão Preto, Universidade de São Paulo, 14040-903 Ribeirão Preto, SP, Brazil; ^3^Laboratório de Pesquisa, Desenvolvimento e Inovação, Apis Flora Industrial e Comercial LTDA, 140120-670 Ribeirão Preto, SP, Brazil; ^4^Núcleo de Pesquisas em Produtos Naturais e Sintéticos, Faculdade de Ciências Farmacêuticas de Ribeirão Preto, Universidade de São Paulo, 14040-903 Ribeirão Preto, SP, Brazil; ^5^Institute of Microbiology, Academy of Sciences of the Czech Republic, Vídeňská, CZ-142 20 Prague, Czech Republic; ^6^School of Chemistry, University of Bristol, Bristol BS8 1TS, UK

## Abstract

Monensin A is a commercially important natural product isolated from *Streptomyces cinnamonensins* that is primarily employed to treat coccidiosis. Monensin A selectively complexes and transports sodium cations across lipid membranes and displays a variety of biological properties. In this study, we evaluated the Jacobsen catalyst as a cytochrome P450 biomimetic model to investigate the oxidation of monensin A. Mass spectrometry analysis of the products from these model systems revealed the formation of two products: 3-*O*-demethyl monensin A and 12-hydroxy monensin A, which are the same ones found in * in vivo* models. Monensin A and products obtained in biomimetic model were tested in a mitochondrial toxicity model assessment and an antimicrobial bioassay against * Staphylococcus aureus, S. aureus* methicillin-resistant, *Staphylococcus epidermidis, Pseudomonas aeruginosa,* and *Escherichia coli.* Our results demonstrated the toxicological effects of monensin A in isolated rat liver mitochondria but not its products, showing that the metabolism of monensin A is a detoxification metabolism. In addition, the antimicrobial bioassay showed that monensin A and its products possessed activity against Gram-positive microorganisms but not for Gram-negative microorganisms. The results revealed the potential of application of this biomimetic chemical model in the synthesis of drug metabolites, providing metabolites for biological tests and other purposes.

## 1. Introduction

**Figure 1:**
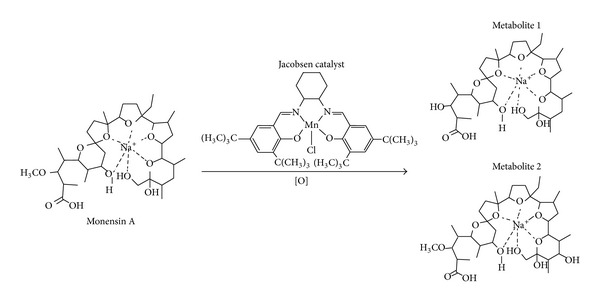
Chemical structures of monensin A, metabolite 1, metabolite 2, and Jacobsen catalyst.

Monensin A (Figure 1) is the main representative drug of the class of polyether ionophore antibiotics of natural origin, isolated from strains of* actinomycetes*. The chemical and biological properties of monensin A are related to their ability to form complexes with cations, especially sodium, and transport the complex formed through cell membranes, thus modifying the normal concentration gradient of Na^+^/K^+^ and thus leading to cell death [1–4].

Since its discovery, monensin A has been widely studied due to its wide spectrum of biological properties such as antimicrobial (especially against Gram-positive bacteria), antiparasitic, antimalarial, and antiviral activities. Furthermore, recent studies on biological properties for cancer therapy have focused on studies of the metabolism of this drug in humans [3–6].

Another important area of concern is the recent reports of the presence of significant levels of waste monensin A in poultry meat and eggs [7–9]. The presence of monensin A in processed food leads to the real eventuality of interaction with other drugs, since monensin A is metabolized by enzymes of the cytochrome P450 3A family (CYP450), resulting in human health problems, such as resistance to antibiotics and poisoning [10–12]. The need to know the actions of monensin A and its metabolites on organisms and the risk of toxicity associated with these compounds exemplifies the need for models that simulate the metabolism of this compound leading to an increased understanding of its mechanism of action, toxicity, and pharmacokinetics in humans.

Cytochrome P450 is a superfamily of enzymes responsible for the oxidative metabolism of a wide variety of xenobiotics in living organisms and they are involved in the metabolism of a wide variety of xenobiotics [13, 14]. In the presence of oxygen donors, Jacobsen catalyst (Figure 1) is known to mimic various reactions of CYP450 enzymes, such as oxidation and oxygenation. Some recent examples in the literature include studies on drugs such as primidone [15], carbamazepine [16], and other synthetic drugs [14], as well as other active natural products such as lapachol [17] and grandisin [18].

*In vitro* studies of drug metabolism using chemical models such as Jacobsen catalyst have several advantages: (1) oxidation products are obtained in relatively large amounts, enabling their use in the identification of* in vivo* metabolites, not to mention the possibility of employing them as standards in pharmacological assays; (2) drug toxicity and action mechanisms can be more easily established; (3) the use of animals for toxicological and pharmacological tests can be reduced [19, 20].

In this context, the aim of this work was to investigate the* in vitro* metabolism of monensin A by applying the Jacobsen catalyst as biomimetical model of CYP450 in order to improve the information available for preclinical pharmacokinetic studies and also to evaluate the toxicity of the products in mitochondrial models compared to monensin A. To conclude, a study of the antimicrobial activity of monensin A and its metabolites was carried out in some Gram-positive and Gram-negative microorganisms in order to evaluate the biological activity of the metabolites generated.

## 2. Materials and Methods

Monensin A (95%) was sourced from Sigma-Aldrich Chemical Co. The Jacobsen catalyst, 3-chloroperoxybenzoic acid (*m*-CPBA), and* tert*-butyl hydroperoxide (*t*-BOOH, 70% solution in water) were all acquired from Acros-Organics. Hydrogen peroxide (30% in water) was supplied by Fluka and stored at 5°C, and it was periodically titrated to confirm its purity. Iodosylbenzene (PhIO) was obtained through iodosyl benzenediacetate hydrolysis and its purity was measured by iodometric assay.

### 2.1. Oxidation Reactions and Isolation of Metabolites

Based on the previous studies by this laboratory [22], the oxidation reactions were performed in an Eppendorf tube (2 mL), under mechanical stirring (Vibrax VXR agitator) at room temperature for 24 h. The ideal molar ratio obtained for the reaction was 1 : 20 : 20 (for catalyst : oxidant : monensin A). This was achieved by adding 0.3 mmol·L^-1 ^: 6 mmol·L^-1 ^: 6 mmol·L^−1^ in the 0.5 mL of reaction medium (CH_2_Cl_2_). The oxidants were* m*-CPBA, PhIO,* t*-BOOH, and H_2_O_2_. The products from monensin A oxidation were analyzed by HPLC-ESI-MS. Control reactions were carried out in the absence of catalyst under the same conditions as the catalytic runs and no products were detected.

The oxidation reaction in preparative scale was performed in a Falcon tube (50 mL), under mechanical stirring (Vibrax VXR agitator) at room temperature for 24 h. The ideal molar ratio obtained for the reaction was 1 : 20 : 20 (for Jacobsen catalyst : PhIO : monensin A). This was 1 : 20 : 20 achieved by adding 0.3 mM : 6 mM : 6 mM in the 25 mL of reaction medium (CH_2_Cl_2_). After that, the separation of products was performed by silica gel preparative TLC using a mixture of CHCl_3_ : MeOH (93 : 7 v/v) as eluent. After elution, the borders of the plates were revealed with solution of vanillin-sulfuric acid (1% vanillin and 1% H_2_SO_4_ in ethanol) for visualization of the metabolites and its further isolation.

### 2.2. Quantification of Monensin A Oxidation by HPLC-ESI-MS Analysis

LC-ESI-MS analyses were performed on a Varian LC-MS 1200L triple quadrupole apparatus coupled to a mass spectrometer with ESI ionization in the positive mode (Varian Medical Systems Inc., Palo Alto, CA). The chromatographic analysis was performed using an injection volume of 10 *μ*L, sample concentration 50 *μ*g·mL^−1^ in an Xterra analytical column MS C-18 (150 × 2.1 mm, 5 *μ*m) (Waters) and following gradient (MeOH : H_2_O): 0.1 min 70% MeOH, 20.0 min 98% MeOH, 21.0 min 30% MeOH, and 30.0 min 70% MeOH. The MS conditions were capillary voltage 3.2 kV, cone voltage 40 V, source temperature 40°C, and N_2_ desolvation temperature 350°C. The percentage of monensin A oxidation was determined by using a calibration curve of 10–100 *μ*g·mL^−1^. The resulting mass spectra were monitored over a *m*/*z* range of 610 to 800.

### 2.3. Identification of Metabolites

The product ion spectra (see Figures S1–S3 in Supplementary Material available online at http://dx.doi.org/10.1155/2014/152102) were obtained using a micrOTOF-Q II hybrid quadrupole time-of-flight (Qq-TOF) mass spectrometer (Bruker Daltonics Inc., Billerica, MA) using positive ion electrospray (ESI) ionization. Monensin A and the metabolites were directly infused into the instrument by syringe pump (Cole-Palmer) at a flow rate of 300 mL·h^−1^. The instrument settings were capillary temperature 250°C, capillary voltage 4.0 kV, and source cone potential 30 V. The nebulizer and drying gas were N_2_ and the collision gas was argon. Tandem mass spectrometry (MS/MS) analysis was achieved on isolated precursor ions using collision induced dissociation (CID) with argon as collision gas at 80 eV.

### 2.4. Antimicrobial Activity Bioassay

The bioassays were carried out using the strains* Staphylococcus aureus *ATCC 25923*, Staphylococcus aureus *ATCC 43300 (MRSA-methicillin resistant),* Staphylococcus epidermidis* ATCC 14990*, Pseudomonas aeruginosa *ATCC 27853,* and Escherichia coli *ATCC 25922 all acquired from the American Type Culture Collection (ATCC). Approximately 5 × 10^5^ microorganisms·mL^−1^ were incubated in Muller Hinton broth in 96-well microtiter plates containing the samples to be tested. The compounds (monensin A, metabolite 1, and metabolite 2) were dissolved in dimethylsulfoxide (DMSO) and diluted into the medium to give 100, 50, 25, 12.5, 6.3, 3.1, 1.6, 0.8, 0.4, 0.2, 0.1, and 0.05 *μ*g·mL^−1^, as their final concentrations. The plates were incubated at 37°C and the bioassays were performed in triplicate. The cell death was determined by a MTT colorimetric method which was described by Andrews [23] and Furtado et al. [24]. The DMSO solution and chloramphenicol were used as controls of the experiment.

### 2.5. Evaluation of Toxicity of Monensin A and Products 1 and 2 Using Mitochondrial Model

Monensin A was diluted in ethanol to concentrations 0.01, 0.1, and 1 *μ*M. All assays were performed in three replicates. Rat liver mitochondria were isolated by standard differential centrifugation according to Pereira et al. [25] and the mitochondrial protein content was determined by the biuret method.

*Mitochondrial respiration* was monitored polarographically with an oxygraph (Hansatech) equipped with a Clark-type oxygen electrode [26]. The mitochondria (1.0 mg protein) were incubated at 30°C in 1 mL of the respiratory medium containing 125 mM sucrose, 65 mM KCl, 10 mM HEPES-KOH, 0.5 mM EGTA, and 10 mM K_2_HPO_4_; pH 7.2. 5 mM glutamate and malate were used as the oxidizable substrates for complex I; and mitochondrial oxidative phosphorylation (state 3) was initiated using 400 nmol ADP.

*Mitochondrial Membrane potential *(Δ_Ψ_) was monitored spectrofluorimetrically using 10 *μ*M safranin-o as a probe in a F-4500 spectrofluorometer (Hitachi) with the 495/586 nm excitation/emission wavelength pair [25]. The mitochondria (1.0 mg protein·mL^−1^) were incubated at 30°C in 2 mL of the standard reaction medium containing 125 mM sucrose, 65 mM KCl, and 10 mM HEPES-KOH, and CCCP (carbonyl cyanide 3-chlorophenylhydrazone) was added at the end of each experiment for the complete dissipation of the membrane potential.

*Mitochondrial swelling* was estimated from the decrease in apparent absorbance at 540 nm performed with a Model DU-70 spectrophotometer (Beckman). The mitochondria were incubated using 30°C in 2 mL of the standard reaction medium.

*Mitochondrial reactive oxygen species (ROS) production* was monitored spectrofluorimetrically with 2′,7′-dichlorodihydrofluorescein diacetate (H_2_DCFDA) with the 503/529 nm excitation/emission wavelength pair, both using the standard reaction medium [27].

### 2.6. Statistical Analysis of Mitochondrial Assays

The experimental data were evaluated by analysis of variance (ANOVA), followed by the post hoc of Tukey, to compare which groups are different from each other and their control using the program GraphPad Prism, version 5.1 for Windows. Results with *P* < 0.05 were considered statistically significant.

## 3. Results and Discussion

Under optimized reaction conditions chosen, the efficiency of the various oxidants PhIO,* m*-CPBA, H_2_O_2_, and* t*-BOOH in the oxidation of monensin A catalysed by Jacobsen catalyst can be measured through the monensin conversion of 36, 27, 26, and 14%, respectively. PhIO was used as an oxygen donor because it is considered as a standard and simple oxidant which contains a single oxygen atom and is well-adapted for the selective and clean formation of metal-oxo intermediates, Mn^V^(O)-salen, more efficient and selective in transferring oxygen to the substrate [13–16]. When using hydroperoxides and peracids as the oxidative agent it is possible for two competing oxygen activation mechanisms to occur. One involves homolytic cleavage of the O–O bond, leading to the formation of the less reactive intermediate Mn^IV^(OH) salen, as well as RO^.^ radicals resulting in reduced yields. The other mechanism involves a heterolytic cleavage of the non-symmetrical O–O bond, leading to the formation of the active specie Mn^V^(O) salen that result in higher yields of oxidation products [13–16]. Considering the monensin A conversion %, the PhIO was chosen as the standard oxidant in order to isolate the oxidative products in preparative scale.

Monensin A oxidation with this biomimetic model leads to the formation of two mains products, product 1 (*m*/*z* 679) and product 2 (*m*/*z* 709). Other reaction media were also tested with little significant influence on the efficiency of oxidation. These two products were isolated by preparative TLC and characterized by ESI-HRMS and MS/MS.

ESI-HRMS was used to determine the molecular formula of these products. The analysis resulted in the [M + Na]^+^ signal at *m*/*z*  679.4008 for product 1 (3-*O*-demethyl monensin A) and *m*/*z*  709,4134 for product 2 (12-hydroxy monensin A) confirming the molecular formula (mass error < 5 ppm) the same as observed for the products obtained from* in vitro* metabolism of monensin A by human liver microsomes and microbial transformation by fungi of* Cunninghamella* genus [21]. Additionally, products 1 and 2 displayed an identical low-resolution product ion spectrum (Supplementary Materials) as well as having the same retention times as those observed in the previous studies [21, 22]. The major ion formed in the MS/MS studies results from a Grob-Wharton type fragmentation and/or H_2_O, followed by CO elimination, as previously proposed for monensin A and its metabolites (Table 1) [21, 22, 28, 29].

Another part of the study was to asses if products 1 and 2 still had toxic activity against the mitochondrial organelle as observed for monensin A. After isolation of the products, they were evaluated to test whether they could damage the mammalian mitochondria. Mitochondria are intracellular structures primarily responsible for transforming the energy from food into useful and transportable energy to the cells through the molecule adenosine 5-triphosphate (ATP). In this way, mitochondria are fundamental to cellular life of most eukaryotic organisms [30]. Since mitochondrial damage may be associated with various tissue injuries or diseases, this organelle has become an important tool for toxicological studies. These studies should help increase the understanding and enable prediction of any adverse effects of various xenobiotics [31, 32]. Often, xenobiotics have different effects on mitochondrial function. Therefore, the use of isolated mitochondria can be considered as a good experimental model to evaluate the toxicological effect of compounds [33, 34].

The results of this study demonstrated that a concentration of only 1 *μ*M for monensin A affected the mitochondrial parameters. Monensin A was observed to increase state 4 of oxygen consumption by 44.11% and consequently decreased the respiratory control ratio (RCR) by 32.59%. It also caused a 47.73% reduction in ADP/O, leading to a decrease mitochondrial respiratory efficiency (Table 2). The products 1 and 2 demonstrated none of these detrimental effects in the study.

**Figure 2:**
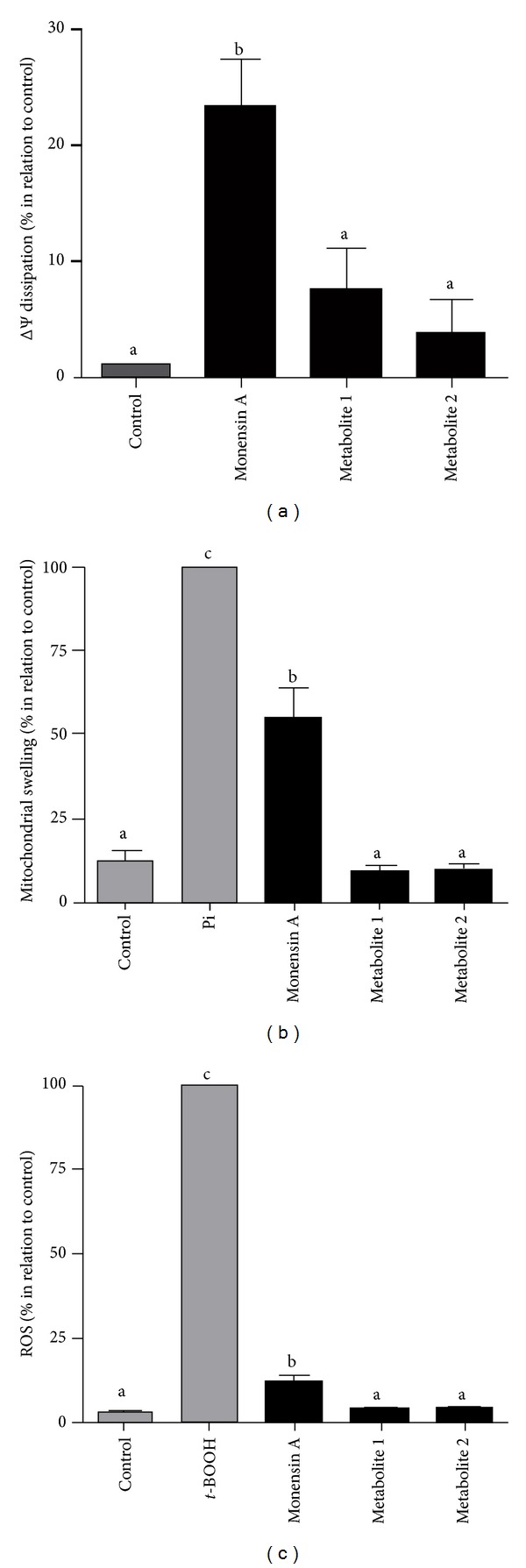
(a) Effect of monensin A (1 *μ*M), metabolite 1 (1 *μ*M), and metabolite 2 (1 *μ*M) on the dissipation of the mitochondrial membrane potential. (b) Effect of monensin A (1 *μ*M), metabolite 1 (1 *μ*M), and metabolite 2 (1 *μ*M) on mitochondrial swelling. (c) Effect of monensin A (1 *μ*M), metabolite 1 (1 *μ*M), and metabolite 2 (1 *μ*M) on mitochondrial production of free radicals. All experiments were performed in mitochondria isolated from rat liver (1.0 mg protein·mL^−1^) incubated as described in Section 2. Points represent the mean ± SEM of three determinations with different mitochondrial preparations, relative to the control in the absence of the compound. *Different letters represent significant differences between treatments according to Tukey's test (*P* < 0.05).

Figures 2(a), 2(b), and 2(c) show the effects of monensin A and its products (1 *μ*M) on mitochondrial membrane potential (*P* = 0.0036), swelling (*P* < 0.0001), and ROS accumulation (*P* < 0.0001), respectively. In this study of these three parameters, significant affects were only observed for the mitochondrial incubated with monensin A.

The increase on mitochondrial state 4 respiration is an indicative of the uncoupler activity of monensin A which leads to the observed decrease of 23.29% on the mitochondrial membrane potential. These effects were probably due to the fact that monensin A has the capacity to cause exchange of H^+^ ions, which is primarily responsible for the formation of the mitochondrial membrane potential [35]. Mitochondrial swelling is also related to the effect on these parameters, reaching 54.61% effect on the apparent turbidity, and it is shown on the literature that monensin A also interferes with the Na^+^ and Ca^2+^ mitochondrial regulation, which affects the osmotic balance causing a swelling in this organelle [36].

Monensin A (1 *μ*M) also affected mitochondrial oxidative stress, increasing by 12.14% accumulation of free radicals, which is in accordance with Ketola et al. [37] which observed the same effect in a strain of prostate cancer cells. The ROS production may occur due to the fact that monensin A caused the deregulation of mitochondrial bioenergetic states arising from ionic alterations, besides having the ability to cause peroxidation of membrane lipids due to the free radical accumulation [38].

It is noteworthy to observe that the products (1 and 2) showed no effect in any of the studied parameters, indicating that metabolism of monensin A prevents its toxic effects to the mitochondria. The toxic effects observed for the interaction of monensin A with isolated rat liver isolated mitochondria can compromise the ATP production as described by Mollenhauer et al. [36]. This occurs because the ATP depletion is one of the early events of compound-induced toxicity resulting from the observed effects on oxygen consumption, dissipation of the mitochondrial membrane potential, and the generation of reactive oxygen species [39, 40]. Other studies about biological activity of product 1 have indicated that it has a much lower antimicrobial, anticoccidial, cardiotoxic, and cytotoxic activity relative to the parent compound [41, 42].

In order to evaluate antimicrobial behavior of monensin A and products 1 and 2, their effects on three Gram-positive and two Gram-negative microorganisms were investigated. The results demonstrated monensin A were efficient against the Gram-positive strains; however, products 1 and 2 presented a reduction in its activity and an inactivation, respectively. These results obtained for monensin A corroborate those in the study by Łowicki and Huczyński [4] that studied monensin A and some semisynthetic analogues (modifications in hydroxyl and carboxyl groups). The semisynthetic esters of monensin A demonstrated antimicrobial action against Gram-positive bacteria, with the results for monensin A against* S. aureus* 25923 being very similar to that presented in this study [2, 4].

Monensin A is extensively metabolized and converted to numerous metabolites by cattle, pigs, and rats.* O*-demethylation and hydroxylation appear to be the major metabolic pathways. Sassman and Lee [42] evaluated antimicrobial activity of* O*-demethyl monensin A by bioautography against* Bacillus subtilis* and by turbidimetric assay against* Streptococcus faecalis. *In these systems 3-*O*-demethyl monensin A was only 5% as active as monensin, suggesting that the monensin is metabolized to products with little or without antimicrobial activity.

The results found for monensin A against* S. aureus* ATCC 25923, 3,1 *μ*g·mL^−1^(Table 3), was practically identical to that one related by Łowicki and Huczyński [4] who found 2.9 *μ*g·mL^−1^ for the same strain, suggesting that the methodology used in this work is reliable. The data found by Łowicki and Huczyński for* S. epidermidis*, however, were different (2.9 and 5.8 *μ*g·mL^−1^ for strains ATCC 12228 and ATCC 35984, resp.) from our results using the same bacteria (Table 3) which can be explained by the different strains used for these authors.

Others studies of the biological activity of product 1 reported on the literature has indicated that this compound has much lower antimicrobial, anticoccidial, cardiotoxic, and cytotoxic activities relative to the parent compound [41, 42], results corroborated by our team, since product 1 presented 25.0 and 50.0 *μ*g.mL^−1^, for* S. aureus* and* S. aureus* MRSA, and product 2 presented >100 *μ*g·mL^−1^.

The biological activity of monensin A depends on the complex formed with cations exhibiting a polar interior and a nonpolar highly hydrophobic exterior which enables free movement across lipid bilayers of cells to exchange cations [3, 4]. This action results in ion imbalance and subsequent biological and toxicological activities [3, 4, 43]. Our results demonstrated that product 1 and product 2 were less active than monensin A for all microorganisms in the conditions studied. Thus, the first step in the metabolism of monensin A is related to the production of more polar metabolites than monensin A, and then, acting like a detoxification step. It can be concluded that the metabolism of monensin A leads to a reduction in the toxicity of this compound in the organism, but that some bactericidal activity for Gram-positive microorganisms remains.

## 4. Conclusion

This work has demonstrated the ability of the Jacobsen catalyst to mimic the action of P450 in monensin A metabolism, with formation of two main products found in the* in vivo* systems: 3-*O*-demethyl monensin A (product 1) and 12-hydroxy monensin A (product 2). The results also revealed the potential of application of this biomimetic chemical model in the synthesis of drug metabolites, providing metabolites for biological tests and other purposes. This was demonstrated in this work allowing interesting information which can help the elucidation of* in vivo* drug metabolism, thus overcoming the difficulty in working with* in vivo* or* in vitro* enzymes systems such as those used in microsomes. The biological tests showed that the products of monensin A have much lower activity or toxicity in all parameters tested. Thus, the first step in monensin A metabolism appears to eliminate or decrease the effects in biological parameters tested and that this effect might be ascribed to greater polarity of product 1 and product 2 that can hamper their transport through membranes.

## Supplementary Material

Supplementary material shows the product ion spectrum of monensin A, metabolite 1 (3-O-demethyl monensin A) and metabolite 2 (12-hydroxy monensin A) obtained in micrOTOF-Q II hybrid quadrupole time-of-flight (Qq-TOF) mass spectrometer using positive ion electrospray (ESI) ionization.

## Figures and Tables

**Table 1:** Major product ions (*m*/*z*) observed in the MS/MS spectra of monensin A and its products (these ions are in accordance with Rocha et al. 2014 [21]).

Monensin A (*m/z*)	Product 1 (*m/z*)	Product 2 (*m/z*)
693	679	709
507	507	523
479	479	495
461	461	477
443	443	459
—	—	441
343	343	343
303	303	303
675	661	691
657	643	673
—	—	655
675	661	691
501	501	517
483	483	499
383	383	441

**Table 2:** Values of the effect caused by monensin A and its products (1 *μ*M) on respiratory parameters.

	V3	V4	RCR	ADP/O
Control	66.107 ± 2.45^a^	9.798 ± 0.32^a^	6.747 ± 0.12^a^	2.874 ± 0.03^a^
Monensin A	64.097 ± 2.35^a^	14.12 ± 0.97^b^	4.548 ± 0.16^b^	1.498 ± 0.01^b^
Metabolite 1	66.363 ± 2.29^a^	10.02 ± 0.11^a^	6.623 ± 0.27^a^	2.757 ± 0.09^a^
Metabolite 2	66.730 ± 1.95^a^	9.832 ± 0.07^a^	6.786 ± 0.15^a^	2.728 ± 0.06^a^

*Respiration rates in nmol O_2_/mg protein/min were performed in mitochondria isolated from rat liver (1.0 mg protein·mL^−1^) incubated.

*Different letters represent significant differences between treatments according to Tukey's test (*P* < 0,05).

*V3 = state 3; V4 = state 4; RCR = respiratory control ratio; ADP/O = phosphorylation efficiency.

**Table 3:** Minimal bactericidal concentration of monensin A, metabolite 1, and metabolite 2 and the controls against *Staphylococcus aureus *ATCC 25923*, Staphylococcus aureus *ATCC 43300*, Staphylococcus epidermidids* ATCC 14990*, Pseudomonas aeruginosa *ATCC 27853, and* Escherichia coli *ATCC 25922. Results are expressed in µg·mL^−1^.

Microorganisms	Samples				
	Monensin A	Product 1	Product 2	DMSO	Chloramphenicol
*S. aureus* 25923	3.1	25.0	>100	>100	6.3
*S. aureus *43300	6.3	50.0	>100	>100	8.3
*S. epidermidis *	25.0	>100	>100	>100	6.3
*E. coli *	>100	>100	>100	>100	3.1
*P. aeruginosa *	>100	>100	>100	>100	100.0
