# Supplementary material for: Jacobsen Catalyst as a Cytochrome P450 Biomimetic Model for the Metabolism of Monensin A
Source: Biomed Res Int. 2014 May 28;2014:152102. doi: 10.1155/2014/152102 (PMC4058456; doi:10.1155/2014/152102)
Supplement: Supplementary file 1 — Supplementary material shows the product ion spectrum of monensin A, metabolite 1 (3-O-demethyl monensin A) and metabolite 2 (12-hydroxy monensin A) obtained in micrOTOF-Q II hybrid quadrupole time-of-flight (Qq-TOF) mass spectrometer using positive ion electrospray (ESI) ionization. [file 152102.f1.pdf]

Supplementary Materials

Jacobsen catalyst as a cytochrome P450 biomimetic model for the metabolism of monensin A

Bruno Alves Rocha,[1] Anderson Rodrigo Moraes de Oliveira,[1] Murilo Pazin,[2] Daniel Junqueira Dorta,[1] Andresa Piacezzi Nascimento Rodrigues,[3] Andresa Aparecida Berretta,[3] Ana Paula Ferranti Peti,[1] Luiz Alberto Beraldo de Moraes,[1] Norberto Peporine Lopes,[4] Stanislav Pospíšil,[5] Paul Jonathan Gates,[6] Marilda das Dores Assis[1*]

Affiliation

[1] Departamento de Química, Faculdade de Filosofia, Ciências e Letras de Ribeirão Preto. Universidade de São Paulo, Ribeirão Preto, Brazil.

[2] Departamento de Análises Clínicas, Toxicológicas e Bromatológicas, Faculdade de Ciências Farmacêuticas de Ribeirão Preto, Universidade de São Paulo, Ribeirão Preto Brazil.

[3] Laboratório de Pesquisa, Desenvolvimento e Inovação, Apis Flora Industrial e Comercial LTDA, Ribeirão Preto, Brazil.

[4] Núcleo de Pesquisas em Produtos Naturais e Sintéticos, Faculdade de Ciências Farmacêuticas de Ribeirão Preto, Universidade de São Paulo, Ribeirão Preto Brazil.

[5] Institute of Microbiology, Academy of Sciences of the Czech Republic (ASCR), Vídeňská, Prague, Czech Republic.

[6] School of Chemistry, University of Bristol, Bristol, United Kingdom

Correspondence

Prof. Dr. Marilda das Dores Assis

Departamento de Química, Faculdade de Filosofia, Ciências e Letras de Ribeirão Preto, Universidade de São Paulo.

Avenida dos Bandeirantes, 3900. 14040-901, Ribeirão Preto, SP, Brazil

Phone: +55-16-3602-0388. Fax: +55-16-3602- 4838.

E-mail: mddassis@usp.br

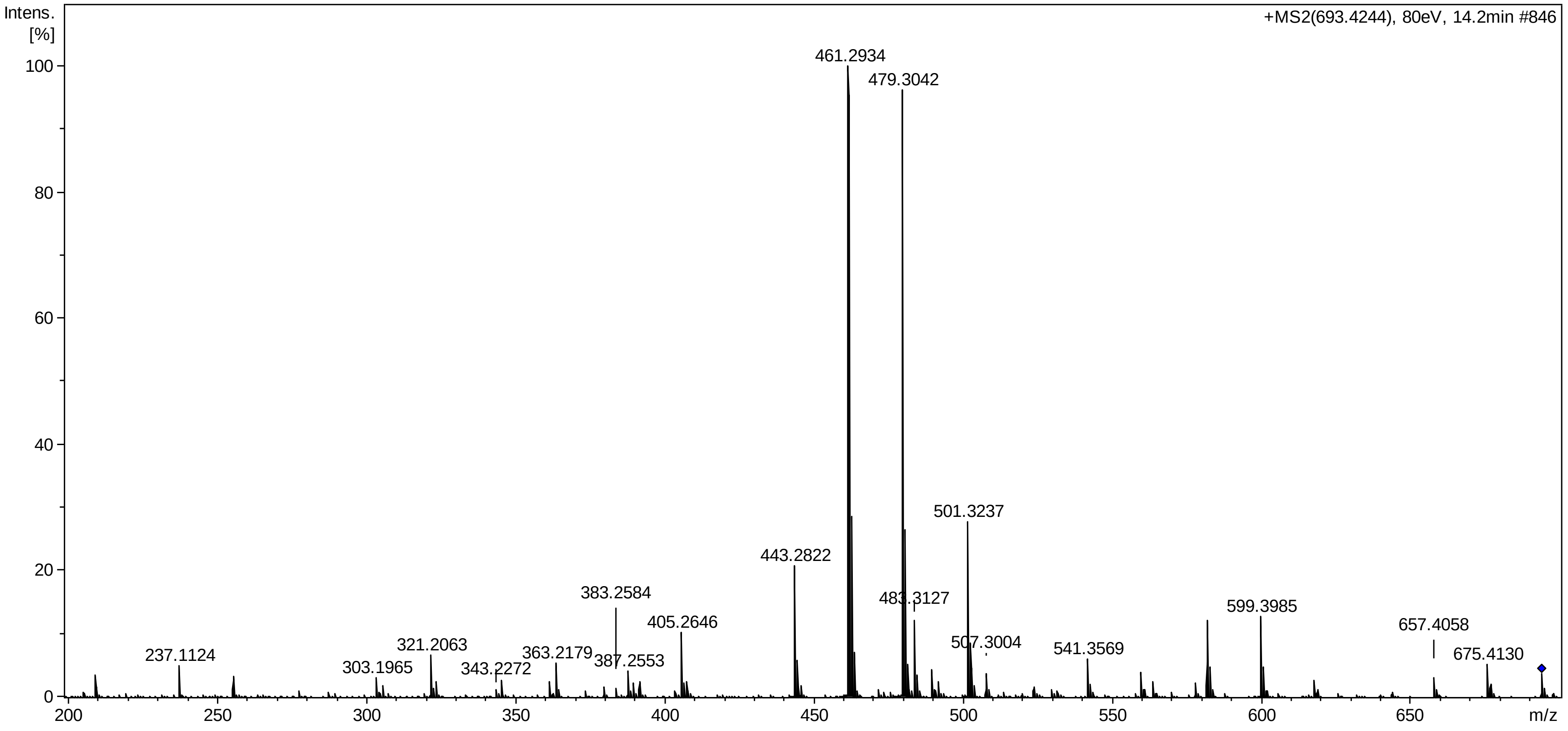

Figure S1: ESI-MS/MS product ion spectrum of monensin A.

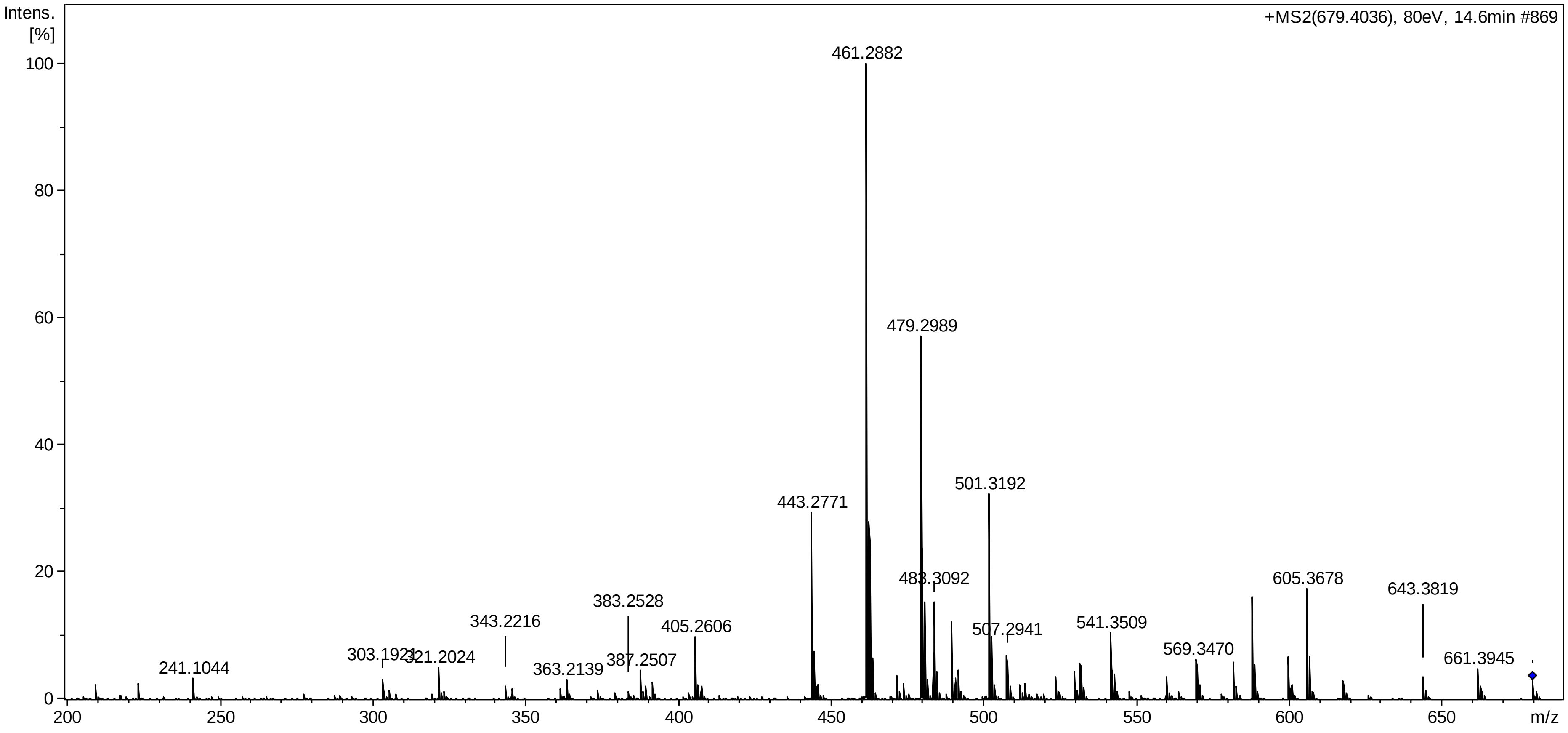

Figure S2: ESI-MS/MS product ion spectrum of metabolite 1 (*m/z* 679)

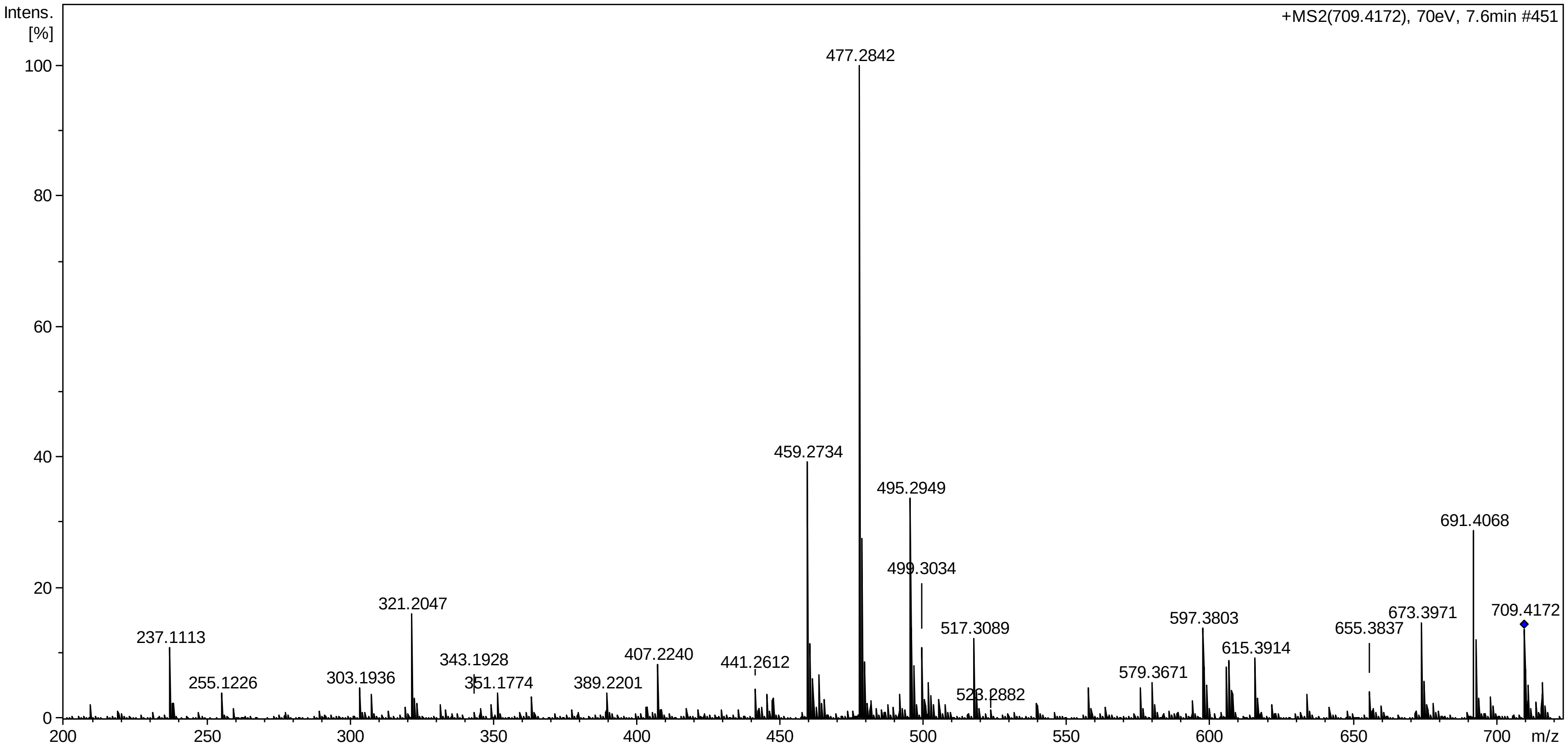

Figure S3: ESI-MS/MS product ion spectrum of metabolite 2 (*m/z* 709).
